# Wide-Awake Hand Trauma Surgery: Designing Strategies to Optimise Patient Experience

**DOI:** 10.7759/cureus.63968

**Published:** 2024-07-06

**Authors:** Alaa Elzagh, Savan Shah, Henry De Berker, Adam J Reid, Jason K Wong, James D Bedford, Kavit R Amin

**Affiliations:** 1 Department of Burns and Plastic Surgery, Manchester University National Health Service Foundation Trust, Manchester, GBR; 2 Department of Otolaryngology, Sheffield Teaching Hospitals National Health Service Foundation Trust, Sheffield, GBR

**Keywords:** carbon footprint, hands first, hand surgery, hand trauma, walant, bssh standards, wide-awake surgery

## Abstract

Introduction: The regional hand trauma service in Greater Manchester, United Kingdom, underwent significant reorganisation early in the COVID-19 pandemic, with a shift from predominantly general anaesthesia (GA) procedures to the adoption of a Wide-Awake Local Anaesthetic No Tourniquet (WALANT) technique. We implemented strategies targeted towards optimising patient experience, largely applicable to most healthcare settings.

Methods: Four domains were explored: (i) compliance in timing to nationally agreed treatment guidelines, (ii) the role of patient information leaflets, (iii) the introduction of a post-operative analgesia protocol, and (iv) broadly evaluating the environmental impact following the implementation of a same-day 'see and treat’ service.

Results: Following reorganisation to a predominantly WALANT service, we observed an increase in compliance with nationally agreed standards for the treatment of common hand injuries. Patient education and peri-operative counselling reduced anxiety, whereas post-operative pain was better managed with the introduction of an analgesic protocol. Using a travel carbon calculator, it can be inferred that there are significant reductions in carbon emissions generated when patients are evaluated and treated on the same day as their clinical presentation.

Conclusions: It is widely acknowledged that WALANT benefits patients and the healthcare system. We contemplated whether further incremental changes in clinical practice could further improve patient experience. Given our findings, we advocate a multi-modal approach with a greater focus on patient outcomes (trials are currently underway, e.g., WAFER) supplemented by universally accepted validated patient-reported outcome measures (PROMs).

## Introduction

Wide-Awake Local Anaesthesia No Tourniquet (WALANT) is an established approach for the management of acute hand injuries. Local anaesthesia (LA, lignocaine and bupivacaine) and adrenaline (1:200000) administered at the site of exploration provide a pain-free, clear field of view that avoids the need for an anaesthetic team and tourniquet [1,2]. We find particular benefits in using this approach when treating high-risk individuals when the safe implementation of general anaesthesia (GA) is in doubt (e.g., multiple comorbidities, pregnancy) [3]. Additional benefits include real-time on-table clinical evaluation following the repair of injured structures. Moreover, intraoperative patient observation of a more ‘functional’ hand in most cases, accompanied by patient education, will likely have a role in improving postoperative compliance with rehabilitation. This may, in part, explain the lower flexor tendon rupture rates associated with WALANT [4,5]. The COVID-19 pandemic was and remains a key driver in shifting the provision of hand trauma practices throughout the UK towards a wide-awake service [6,7]. A large tertiary hand trauma service in London estimates cost savings to be as high as 70% [8].

Based in Greater Manchester, United Kingdom, we serve a population of 2.8 million and undertake approximately 2500 acute hand trauma procedures annually. We previously reported our early experiences in service reorganisation towards WALANT in the peri-COVID period [6]. Not only did this increase service capacity, but patients also perceived they had a positive experience [9]. An audit of our discharge summaries (using criteria set by the Royal College of Surgeons and Professional Record Standards Body; n = 140) [10] provided us with a set of service improvement goals. For example, postoperative care information was limited, including aftercare, the importance of elevation, antibiotics when relevant, and how best to control postoperative pain. This body of work aimed to devise and analyse strategies that address such key features in the successful management of acute hand injuries and crucially, whether the standards of care set by the British Society for Surgery of the Hand (BSSH) for timing to surgery were being accomplished.

## Materials and methods

Approval and registration for data collection were granted via the Clinical Audit Department of Manchester University National Health Service (NHS) Foundation Trust, Manchester, United Kingdom. Four domains were evaluated (Table 1), with data sourced from a total of 2400 patients. These domains were generated in response to our audit on the accuracy of discharge summaries and feedback from patients and staff as well as data published from our unit [9].

**Table 1:** Four interventional domains investigated. BSSH: British Society for Surgery of the Hand; GA: general anaesthesia; WALANT: Wide-Awake Local Anaesthesia No Tourniquet

Domain	Sample	Project summary	Statistics (Significance p<0.05)
(1) Time to treatment	900	Standards set by the BSSH for the time to treatment of commonly encountered presentations (extensor/flexor tendon, nerve, open/closed fractures, revascularisation/replantation) were analysed [11]. Two parallel six-month periods were compared a year apart (September-February) with data evaluated pre- (primarily GA clinical practice) and post-pandemic (primarily WALANT to mitigate the risk of COVID-19 spreading). Group I: (Pre-pandemic) Six months in the lead-up to the first lockdown (September 2019-February 2020). Group II: (Pandemic) (September 2020-February 2021).	Between-group chi-squared test
(2) Pre/post-operative leaflet	40	Patient information leaflets (PILs) were designed using ‘best practice guidance in the delivery of patient information’ [12]. A leaflet was given to patients in the trauma clinic informing them about what to expect on the day of surgery, answering queries about pain and analgesia, and reporting on the benefits and disadvantages of WALANT as an alternative to GA/regional block. A questionnaire was designed to capture knowledge both prior to and after reading the leaflet with scores recorded using a modified Likert scale.	Between-group chi-squared test
(3) Post-operative analgesia	60	Patient feedback directed us towards developing a standardised approach to address post-operative pain. Patients were contacted by telephone on days one and seven to score pain using a visual analogue scale (VAS). Cycle 1 involved assessing whether pain was adequately managed whilst cycle 2 assessed the efficacy of multi-modal analgesia with a concurrent protocol with instructions for administration.	Student’s t-test
(4) Carbon footprint	1400	Estimating the impact of WALANT ‘same day treatment’ on carbon footprint	Between-group chi-squared test

## Results

Time to treatment

Similar numbers of presentations were managed in both cycles (451 in group I vs. 449 in group II), and largely in compliance with national standards both prior (75.4%; primarily GA) and during the pandemic (78.86%; primarily WALANT) (Table 2).

**Table 2:** Procedures performed in both groups under LA and GA.

Category	Pre-pandemic	Pandemic
Number of procedures	451	449
Local anaesthesia (LA)	153 (33.92%)	382 (85.08%)
General anaesthesia (GA) support	298 (66.08%)	67 (14.92%)

The subgroup analysis revealed a significant increase in compliance for extensor tendon repair (pre-pandemic: 68.97%; pandemic: 85.94%, p = 0.042) (Table 3).

**Table 3:** BSSH Standards of Care in Hand Trauma time-to-treatment target. p < 0.05 is considered significant. BSSH: British Society for Surgery of the Hand

Category	BSSH targets	Compliance (pre-pandemic)	Compliance (pandemic)	p-value (chi-square/Fisher’s exact)
Flexor tendon repair	Four days	82/104 (78.85%)	90/115 (78.26%)	0.953
Extensor tendon repair	Four days	40/58 (68.97%)	55/64 (85.94%)	0.042
Nerve repair	Four days	75/109 (68.81%)	97/131 (74.05%)	0.452
Closed fractures	Seven days	119/126 (94.44%)	84/94 (89.36%)	0.254
Open fractures	24 hours	17/45 (37.78%)	13/32 (40.63%)	0.988
Revascularisation of digit	24 hours (digit)	7/9 (77.78%)	13/13 (100%)	0.471
Total		340 (75.39%)	353 (78.62)	0.284

Preoperative information leaflet

A significant improvement across all knowledge-based categories was observed (Table 4).

**Table 4:** Data comparing patient knowledge of WALANT following the implementation of a PIL. p < 0.05 is considered significant. WALANT: Wide-Awake Local Anaesthesia No Tourniquet; PIL: patient information leaflet

Questions	Pre-leaflet	Post-leaflet	p-value
I understand better what WALANT is and what it is used for.	2.50%	97.50%	<0.001
I am prepared for the discomfort WALANT may cause and understand how long it lasts.	7.50%	95%	<0.001
I am aware of the benefits of WALANT over general anaesthesia.	5%	97.50%	<0.001
I understand the potential adverse effects of WALANT and which medications I need to stop taking prior to the procedure.	2.50%	97.50%	<0.001
I am more aware of when to return to my normal activities (driving, handling, etc.)	5%	95%	<0.001

Postoperative analgesia regimen

Cycle 1 (n=32) demonstrated mean pain scores (maximum=10) in the immediate postoperative period of 0.19/10, day one (5.91/10), and day seven (2.16/10), indicating a peak on the first postoperative day (Table 5). The majority of patients taking codeine (n = 14, 70%) were not co-administered paracetamol and ibuprofen. Analgesia was taken by 43.75% of individuals.

**Table 5:** Cycle 1 pain scores across patient groups. M: mean; SD: Standard deviation

Group	N	Day one pain scores	Day seven pain scores
All participants	32	(M = 5.91, SD = 2.43)	(M = 2.16, SD = 1.87)
Codeine	20	(M = 5.70, SD = 2.54)	(M = 2.10, SD = 1.80)
No Codeine	12	(M = 6.25, SD = 2.30)	(M = 2.25, SD = 2.05)

In cycle 2 (n=30), all patients were provided with codeine on discharge and provided with a written protocol with encouragement to also take paracetamol and ibuprofen. Patients reported high scores on the same evening of surgery (mean (M) = 6.60) (Table 6). A third of individuals reported pain affecting sleep and approximately 7% sought hospital input. However, average day-one pain scores reduced to 3.34 with regular codeine administration and patient information leaflet (PIL) guidance, demonstrating statistical significance compared with cycle 1 (p<0.00001). Codeine is therefore sufficient alongside paracetamol and ibuprofen with particular emphasis placed on targeting high-risk groups (eg., cognitive or renal impaired people).

**Table 6:** Cycle 2 pain scores. M: mean; SD: standard deviation

Group	N	Day zero (evening) pain scores	Day one pain scores
All participants	30	(M = 6.60, SD = 2.83)	(M = 3.34, SD = 1.79)

Impact of WALANT on carbon footprint

The NHS accounts for 4% of the total carbon footprint in England with 5% of emissions contributed by patient travel [13]. This highlights the growing need for all services to innovate and develop strategies that target reductions in carbon emissions [14]. Our regional centre (population: 2.8 million, regional area: 1,277 km2) implemented a same-day ‘see and treat’ service. Of the 1400 patients treated in the pandemic (nine months), twenty percent were suitable for this service. Assuming patients travel from their home postcode, this represents an average reduction in travel of 40 km per patient (11,100 km collectively). Using a travel carbon calculator, the first 10 months after setting up the service generated an estimated saving of 1.6 tonnes of CO2. This represents an annual saving of 1.9 tonnes of CO2.

## Discussion

The development of our WALANT service was already in transition before the pandemic but accelerated to ensure patients and staff were protected from COVID-19. Following this rapid restructuring and deployment, we felt it was justified to address whether WALANT influenced compliance with national guidelines for time to treatment. Comparable levels of compliance with national standards both pre- (predominantly LA) and post-pandemic (predominantly GA) were observed. The pandemic provided a range of challenges to the delivery of healthcare services both nationally and internationally. The learning curves during the increasing introduction of WALANT and shared international experiences warrant further investigation. Launched in 2023, post-dating the data collection in this article, the HandsFirst2 initiative is now actively recruiting trusts across the UK, drawing insights from the Hands First QI Collaborative. The primary objective is to ensure that 80% of hand injuries requiring surgery undergo their initial operation within specified timeframes, aligning with the BSSH standards for hand trauma. This commitment is geared towards enhancing patient care, minimizing complications, and potentially yielding cost savings for the involved trusts and health boards [15]. The critical finding that can be stated from our data is the lesser reliance upon GA and anaesthetic support teams. We implemented a 'see and treat’ service adjoined to our WALANT site, which significantly reduced waiting times and optimised service delivery by using a separate theatre suite. This has been similarly acknowledged at other centres [16,17]. This serves as an excellent training opportunity for junior surgeons and complements previously published reports [9].

We identified that a preoperative information leaflet significantly improves patient knowledge and alleviates anxiety [18]. Tom et al. found a video resource to be a useful adjunct in supplementing preoperative education and improving psychological and physical well-being [19]. We have since produced a video to supplement our leaflet (quick-response code (QR)-code activated), informing patients about what to expect on the day of surgery. Post-operative patient information has been helpful for both nursing staff, patients, and their families. Renna et al. reported the effectiveness of a similar post-operative leaflet for lower limb surgery [20]. They similarly observed high satisfaction levels having addressed time to recovery, wound care, and postoperative pain. Further advice was provided about driving, sports, return to work, and how to access clinical support from our team.

The consideration of post-operative pain is crucial for any medical intervention. This facilitates patient engagement, enhances recovery, and mitigates re-presentation to healthcare providers. We identified a widespread knowledge gap in postoperative analgesia administration amongst patients and appropriately addressed this through a standardised protocol and supplemental written guidance. The efficacy of multi-modal analgesia [21,22] and the importance of regular administration are well established [23]. Moreover, although patients are advised to keep their arms elevated to reduce oedema, we intend to make this a feature in our revised leaflet and video, given that the benefits of limb elevation in controlling oedema are well established [24]. We are currently in the process of seeking formal approval to dispense a limited supply of stronger opiates to lessen the burden placed on primary and emergency care services for selected individuals.

The anticipated environmental benefits of WALANT have allowed us to consider our team's role in social responsibility in relation to climate change. Decarbonisation via ‘same day see and treat’ when possible (reduction in patient travel), and streamlining surgical kits for autoclaving and surgical draping are some of the surgical innovations impacting our environment. More intriguing is the diminishing reliance upon anaesthetic agents. The relationship between climate change and human health led the NHS, the first universal healthcare systems provider in the world, to pledge a net zero healthcare system by 2040 [13]. Anaesthetic gases comprise 2% of greenhouse gas emissions from the NHS [25]. Agents such as desflurane have potent global warming potential (over 20 years, a single 240 ml bottle is equivalent to 1296 kg of CO2) [26]. A single hour of anaesthesia with desflurane in a 70 kg patient contributes over 5.5 kg of CO2 [27]. The NHS Long Term Plan is committed to reducing anaesthetic gas consumption by 40% and is one of the reasons total intravenous anaesthesia (TIVA) was introduced. The 77% reduction in GA within our department (from 66% to 15%) demonstrates that this is a critical area for research given the commitment to environmental reform amongst hand surgeons globally [28]. Future efforts must focus on evaluating patient-reported outcomes and experience and targeting how to best enhance staff experience in delivering a WALANT service.

Limitations

There were limitations to this study, particularly in drawing any firm conclusions from pre-pandemic and pandemic data. Factors such as the nature of injuries, surgeon learning curves, and delay in presentation are all potential confounding factors. Moreover, the lack of comprehensive patient-reported outcome measures (PROMs) poses a challenge in fully capturing patient perspectives and experiences from WALANT. Similarly, assessing the environmental impact of WALANT on the carbon footprint is a complex problem to fully quantify but warrants further investigation. Collectively, these limitations emphasise the need for ongoing research into the benefits, improvements and implementation of WALANT services.

## Conclusions

The pandemic provided an opportunity to evaluate our transition into a primarily WALANT service. Despite this swift adjustment in practice, we have been successful in maintaining and improving our compliance with BSSH standards. The introduction of a PIL, protocols for how best to administer analgesia, and the potential financial cost savings and beneficial impact on the environment arguably favour WALANT as the optimal future strategy for the management of hand trauma victims. This is well aligned with the strategies set by the BSSH.
